# Effects of Initial Cell Density and Hydrodynamic Culture on Osteogenic Activity of Tissue-Engineered Bone Grafts

**DOI:** 10.1371/journal.pone.0053697

**Published:** 2013-01-11

**Authors:** Fei Luo, Tian-Yong Hou, Ze-Hua Zhang, Zhao Xie, Xue-Hui Wu, Jian-Zhong Xu

**Affiliations:** Department of Orthopedics, Southwest Hospital, The Third Military Medical University, Chongqing, China; New Jersey Medical School, University of Medicine and Dentistry of New Jersey, United States of America

## Abstract

This study aimed to study the effects of initial cell density and *in vitro* culture method on the construction of tissue-engineered bone grafts and osteogenic activities. Human mesenchymal stem cells (hMSCs) were seeded onto cubic scaffolds prepared from demineralized bone matrix (DBM) by three methods - static, hydrodynamic, or fibrin hydrogel-assisted seeding. The resulting cell-scaffold constructs were cultured *in vitro* by static flask culture or hydrodynamic culture. The initial cell density and the subsequent *in vitro* proliferation and alkaline phosphate activities of the constructs were analyzed. The constructs were also subcutaneously implanted in nude mice to examine their *in vivo* osteogenic activities. Hydrogel-assisted seeding gave the highest seeding efficiency, followed by hydrodynamic and conventional static seeding. During *in vitro* culture, hydrodynamic culture produced higher plateau cell densities, alkaline phosphatase (ALP) activities, and extracellular matrix production than static culture. After subcutaneous implantation in nude mice, the implants prepared by the combination of hydrogel-assisted seeding and hydrodynamic culture produced higher wet weight and bone mineral density than implants prepared by other methods. The results suggest that the hydrogel-assisted seeding can substantially increase the initial seed cell density in scaffolds. Subsequent hydrodynamic culture can promote the proliferation and osteoblastic differentiation of the seeded cells. Correspondingly, bone grafts produced by the combination of these two methods achieved the highest osteogenic activity among the three methods employed.

## Introduction

Reconstruction of critical-size bone deficiencies remains a major challenge in orthopedics. The bone tissue engineering technique provides a new approach to this problem [1], [2], [3], [4]. The seeding and subsequent *in vitro* culture fundamentally affects the osteogenic activity of tissue-engineered bone grafts [3] because they determine the initial density and spatial distribution of seeded cells in the scaffold as well as their subsequent behaviors (e.g. proliferation, differentiation, migration) [1], [2], [4]. Many factors can affect the efficiency of seeding and the outcome of the subsequent *in vitro* culture, including in the technique employed for seeding and the hydrodynamic condition provided for subsequent regeneration [5], [6].

Currently, cells are seeded primarily by static or hydrodynamic methods. In the static method, a suspension containing seeded cells is dispensed on a scaffold, followed by a period of rest to allow the cells to enter the scaffold. With this method, the initial cell density (the number of cells which attached in 3D scaffold when tissue engineering bone were preparation and without culturing in vivo or in vitro) in the scaffold can be increased by increasing the cell concentration of the suspension within a certain range, though at the expense of seeding efficiency (i.e. the percentage of cells that entered the scaffold), but cannot be further increased beyond a plateau level [6]. In comparison, in the hydrodynamic seeding method, cells are allowed to adhere to the scaffold in a dynamic fluid flow created by a bioreactor. With this method, the cell agglomeration accelerates with the cell density in the seeding suspension, thus facilitating the adherence of cells to the scaffold, increasing the speed and density of cell seeding, and improving the spatial distribution of cells in the scaffold [7], [8]. In addition to seeding, hydrodynamic conditions can also substantially affect the subsequent *in vitro* culture of cell-scaffold constructs. A dynamic fluid flow was found to positively affect the behavior of seeded cells, such as proliferation, differentiation, and migration [4], [7], [9], [10], [11]. However, dynamic fluid flow may also result in cell detachment and shear-induced damage, and thus, loss in cell utilization [3], [12].

A number of studies have separately exploited the advantages associated with a higher initial cell density or hydrodynamic culture [7], [13]. Zhao *et al* increased the initial density of human umbilical cord mesenchymal stem seeded cells in injectable bone tissue engineering constructs by using hydrogel microbeads [13]. Ericka *et al* seeded chondrocytes onto polyglycolid acid scaffolds under hydrodynamic conditions, and obtained intermediate initial cell densities and sustained subsequent proliferation [7]. The optimal tissue engineering technique should combine methods to increase the initial cell density and create an appropriate hydrodynamic environment to accelerate the *in vitro* maturation of the cell-scaffold constructs into clinically applicable grafts.

Here, we investigate whether a combination of fibrin glue-assisted seeding and hydrodynamic culture in rotating wall vessel bioreactor can substantially improve the seeding efficiency and subsequent proliferation and osteoblastic differentiation. We further determined if these improvements translated into enhanced osteogenic activity in a nude mice subcutaneous implantation model. This study aims to understand the effects of the key factors of tissue engineering preparation methods, including initial cell density and hydrodynamic culture methods, in an attempt to provide experimental basis for improvement the osteogenesis performance of bone tissue engineering.

## Materials and Methods

### Ethics statement

Nude mice (6 weeks old) were purchased from the Laboratory Animal Center of our university. The animal experiment was approved by the ethics committee of Third Military Medical University and conducted in conformity with the ‘Guiding Principles for Research Involving Animals and Human Beings’ as adopted by The American Physiological Society.

### Isolation and characterization of hMSCs

Human mesenchymal stem cells (hMSCs) derived from bone marrow of the iliac crests of young healthy volunteers were provided from Tissue Engineering Research and Development Center of The Third Military Medical University. hMSCs were isolated by density gradient centrifugation in Percoll (density = 1.077 g/mL; Sigma, USA) and adherent culture. The cells were expanded by in vitro culture and confirmed to be hMSCs by a previously published method [14]. To induce osteogenic differentiation, the hMSCs were cultured in DMEM/F12 medium supplemented with 10% FBS, 0.1 µM dexamethasone, 10 mM glycerophophate, and 50 µM ascorbic acid [15], [16]. After 12 days, the induced cells were detected by immunochemistry using antibodies (1∶100) for ALP, osteocalcin and collagen type I (Sigma, USA) according to product instructions [17]. At the 12th day after osteoinduction, the ALP activity of induced hMSCs was measured and the osteocalcin concentration in the culture medium of induced hMSCs was detected by an enzyme-linked immunosorbent assay (ELISA) kit (BioSino, Beijing, China).

### Scaffold preparation

Cubes of human demineralized cancellous bone matrix (DBM, 4 mm×4 mm×4 mm) were obtained from the tissue bank of our university and used as the scaffolds in this study. The porosity of DBM is 70% and the pore size is 300–800 µm. The DBM was prepared by a series process, as previously described by Tan et al. [18].

### Construction of implants and Grouping

Four kinds of bone substitutes were constructed based on different approach of seeding and culture (Table 1).

**Table 1:** Summary of in vitro preparation of cell-scaffold constructs.

Group	Seeding method	Culture condition	Cell suspension concentration per scaffold	Cell suspension volume per scaffold	Cell number per scaffold	Seeding efficiency (%)
A	Hydrodynamic	Hydrodynamic	1.0×10^6^ cells/ml	1.00 ml	1.0×10^6^	32.10±0.72#
B	Hydrogel-assisted	Hydrodynamic	2.0×10^7^ cells/ml	0.05 ml	1.0×10^6^	82.14±1.09
C	Static	Static	2.0×10^7^ cells/ml	0.05 ml	1.0×10^6^	25.24±1.56*
D	Hydrogel-assisted	Static	2.0×10^7^ cells/ml	0.05 ml	1.0×10^6^	81.53±1.37

* : seeding efficiency in group C was lower than that in other groups (*p<*0.05);

# : seeding efficiency in group A was lower than that in group B and D (*p<*0.05).

Hydrodynamic seeding and hydrodynamic culture (group A): Fifty DBM scaffolds and 5.0×10^7^ MSCs were added into the high-aspect ratio vessel of a rotary cell culture system (Synthecon RCCS-1, Houston, TX, USA). The vessel was filled with 50 ml of DME/F12 culture medium (Hyclone, Logan, UT, USA) and degassed. The rotation speed was adjusted daily (18–24 rpm) to ensure that the rotating trajectories of the scaffolds would not collide with the vessel wall or converge to the center. The rotary culture system was incubated in an atmosphere of 5% CO_2_ and 100% relative humidity at 37°C, with daily adjustment of rotation speed and a change of medium every 48 h.

Hydrogel-assisted seeding and hydrodynamic culture (group B): The fibrin glue (25 mg/ml, Tissucol, Baxter, Austria) was prepared by mixing fibrinogen and thrombin within 90 min under sterile condition. The hMSCs were mixed with a fibrin glue to form a suspension containing 2×10^7^ cells/ml. The suspension was added dropwise onto the DBM scaffolds (0.05 ml/scaffold). Then 0.05 ml of catalytic agent was sprayed on each scaffold, and was allowed to stand for 5 min for the glue to gelate. Then the scaffolds were added into the high-aspect ratio vessel of a rotary cell culture system, and were cultured with the same condition of group A.

Static seeding and static culture (group C, control group): The hMSCs were dissociated in DMEM/F12 medium into a suspension containing 2×10^7^ cells/ml and added dropwise onto the DBM scaffolds (0.05 ml/scaffold). The seeded scaffolds were statically cultured in 24-well plates without additional medium for 2 h, then turned over to reduce the downward flow of liquid and the cell loss, and cultured in the same conditions for another 2 h. Then, each well was filled with 1 ml DMEM/F12 and incubated in an atmosphere of 5% CO^2^ and 100% relative humidity at 37°C, with a change of medium every 48 h.

Hydrogel-assisted seeding and static culture (group D): DMB scaffolds were seeded by the same hydrogel-assisted method as in the group B and were cultured statically in 24-well plates (Corning 3524, USA) containing DME/F12 medium (1 ml/well) and incubated in an atmosphere of 5% CO_2_ and 100% relative humidity at 37°C, with a change of medium every 48 h.

### Evaluation of seeding efficiency

Twenty four hours after seeding, the seeding efficiency of each group was analyzed, which was defined as the ratio of the number of cells existing in the scaffold to the number of cells added originally to the scaffold. After transferring bone substitutes to other wells, non-adherent cells in the well were collected by rinsing repeatedly with DMEM/F12 medium. Then, the cells attached to well bottom were digested with 0.25% trypsin plus 0.01% EDTA and collected. The cells in the supernatant and those adhering to the bottom of the well were separately counted by hemocytometer. The sum of these two portion of cells was recorded as ‘remaining cell number’ in each well. The seeding efficiency was calculated by: (initial cell number-remaining cell number)/initial cell number.

### Cell viability

The viabilities of cells in scaffolds were assayed at various time points (8 h, 16 h, 24 h, 48 h, and 3–14 d after seeding). The cell-scaffold constructs were removed from their medium, rinsed with phosphate buffered saline (PBS), and placed in a 96-well culture plate. Cell viability was determined as reported [19]. Cell counting kit-8 (CCK-8, 20 µL/well, Dojindo Chemical Institute, Kumamoto, Japan) was added to each well, followed by further culture for 3 h (5% CO 2, 37°C, 100% relative humidity). Then, the constructs were removed, and the optical density of each well at 450 nm was measured with an ELISA reader (reference wavelength: 655 nm), with cell-free DMB scaffolds as the controls.

### ALP activity

The ALP activities were measured at various time points (2, 4, 6, 8, 10, 12, 14 and 16 d) after seeding. The cell-scaffold constructs were rinsed twice with PBS and then lysed with 0.2% Triton X-100 (Sigma, USA). The lysate was centrifuged at 600 g for 5 min and the supernatant was collected and incubated for 15 min (5% CO_2_, 37°C, 100% relative humidity). The absorbance at 405 nm was measured on a microplate reader and converted into the ALP activity against a standard curve, which was established based on the reaction of 10 ml of a p-nitrophenyl solution (Wako) and 200 ml of substrate buffer for 30 min. ALP activities were expressed as U values.

### Scanning electron microscopy (SEM)

All specimens per group were was treated by a series of procedures for SEM observation after 14-day culture, including incubating in 3% (w/v) glutaraldehyde solution for 48 h at 4°C, washing over-night with 0.1 M PBS solution, decalcifying with 0.5 mol/L EDTA for 7 days, washing over-night with 0.1 M PBS solution, incubating in 2.5% glutaraldehyde solution for 48 h at 4°C, and post-fixing and staining with 1% osmium tetroxide for 2 h at 4°C, dehydrating in a graded series of ethanol, coating with an ultrathin gold layer. Then the specimens are observed under SEM (1000-B, AMRAY, Bedford, MA, USA) to assess investigate morphological features of the attached cells and ECM fibers formed on scaffolds.

### In vivo osteogenesis

The *in vivo* osteogenetic activities of scaffolds and cell-scaffold constructs were evaluated with a subcutaneous implantation model in 24 nude mice. Each mouse received four implants in its back, termed implants I–IV (Table 2). Implant I was a cell-free DBM scaffold and placed at the left rostral position. Implant II was a cell-scaffold construct seeded with 2×10^7^/ml hMSCs by the hydrogel-assisted method followed by dynamic culture for 12 d; this was placed at the right rostral position. Implant III was a construct seeded with 1×10^8^/ml hMSCs by the hydrogel-assisted method; it was placed at the left caudal position immediately after seeding without *in vitro* culture. Our pretest found that during the 12-day dynamic culture (as in Implant II), cell proliferation in the scaffold increased by 10 fold (data not shown). Therefore, to ensure an equal cell density before *in vivo* implantation, the initial cell density for implant III was 9 times greater than implant II. Implant IV was a construct seeded with 2×10^7^/ml hMSCs by the hydrogel-assisted method followed by static flask culture for 12 d; it was placed at the right caudal position.

**Table 2:** Implant groups for subcutaneous implantation.

Group	Scaffold	Seeding condision	Cell number per scaffold	Culture time	Place in nude mouse
I	DBM	no	no	no	left rostral
II	DBM	Hydrogel-assisted	1.0×10^6^	dynamic culture 12 d	right rostral
III	DBM	Hydrogel-assisted	1.0×10^7^	no	left caudal
IV	DBM	Hydrogel-assisted	1.0×10^6^	static flask culture 12 d	right caudal

The mice were killed by decapitation 4, 8, and 12 weeks after implantation (n = 8, for each time point). Each mouse was placed in a supine position and examined by X-ray radiography. The implants were retrieved, stripped of soft tissues, and weighted wet. They were also analyzed by dual-energy X-ray absorptiometry (Challenge, DMS, Montpellier, France) for bone mineral densities.

### Histological analysis

Histological analysis of the implants was undertaken at 12 weeks after surgery. Specimens from the bone defect sites were fixed with 4% paraformalclehyde for 48 hours, decalcified with 0.5 mol/L EDTA for 2 weeks, dehydrated with gradient ethanol solutions for 2 days, vitrified with dimethylbenzene, embedded in paraffin, and cut to yield 6 µm thick sections. The sections were stained with haematoxylin and eosin (H&E) for histological evaluation and examined under light microscope.

### Statistical analyses

Data were expressed as mean ± standard deviation. Data were analyzed by one-way analyses of variance (ANOVA) and Student–Newman–Keuls (SNK) post hoc tests using SPSS 12.0 (SPSS, Chicago, IL, USA). A p-value of less than 0.05 was considered statistically significant.

## Results

### Cell culture and characterization

**Figure 1:**
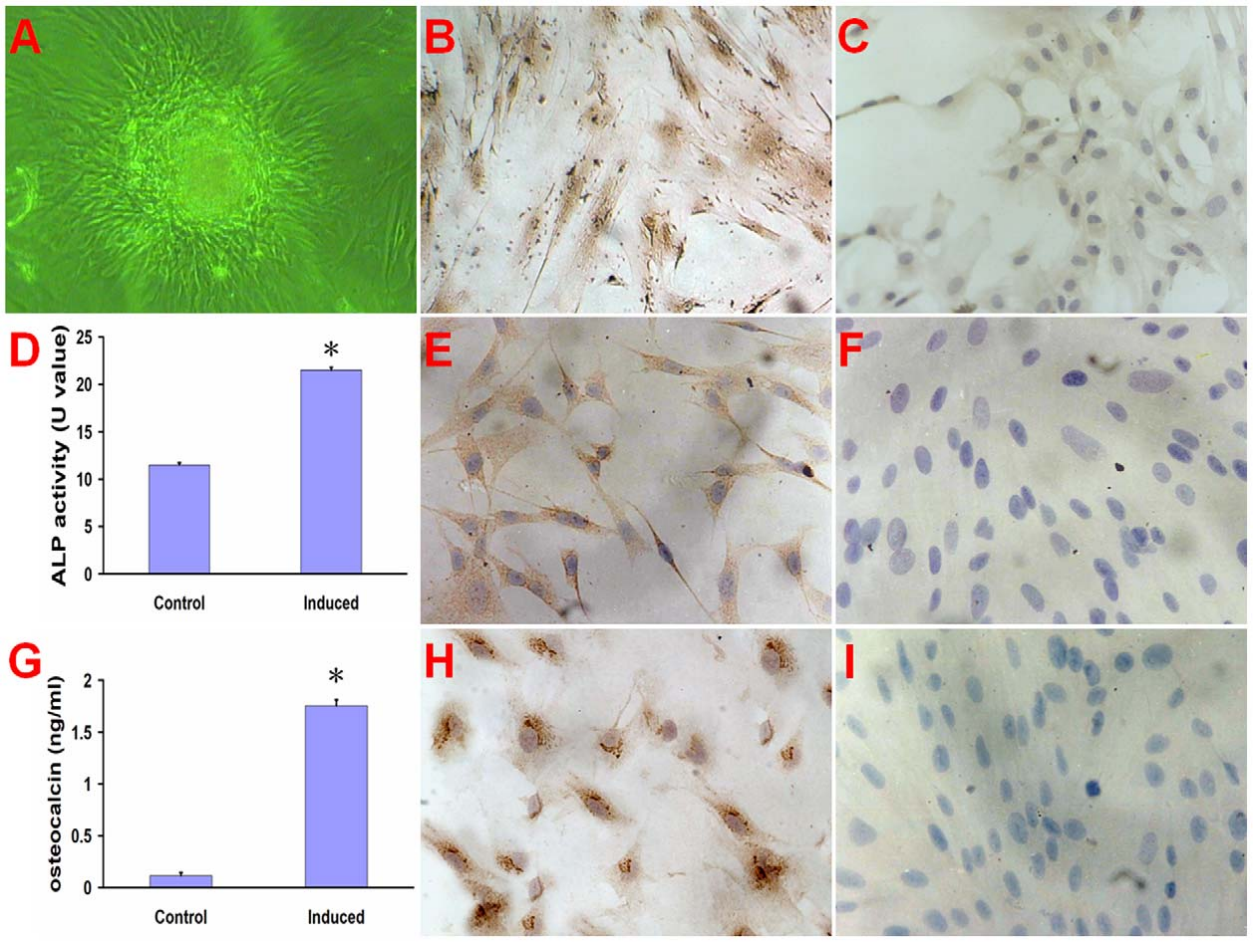
The culture and characterization of hMSCs. The hMSCs formed calcium nodus after 12 day culture (A). The hMSCs (200×) stained immunohistochemically positive for ALP (B), osteocalcin (E) and collagen type I (H) compared to non-induced cells (C, F, and I). The ALP activity (D) of hMSCs and osteocalcin concentration (G) in the culture medium were significant higher in induced group than that in control group (non-induced cells). *p < 0. 01, compared with control group.

The hMSCs tended to form calcium nodus after 12 d conditional culture (Fig. 1A). The hMSCs also stained immunohistochemically positive for ALP (Fig. 1B and C), osteocalcin (Fig. 1E and F) and collagen type I (Fig. 1H and I) after 12-day osteogenic induction. The ALP activity of hMSCs (Fig. 1D) and osteocalcin concentration (Fig. 1G) in the culture medium were significantly higher in induced group than that in control group (P<0. 01).

### Microscopy of cell-scaffold constructs

**Figure 2:**
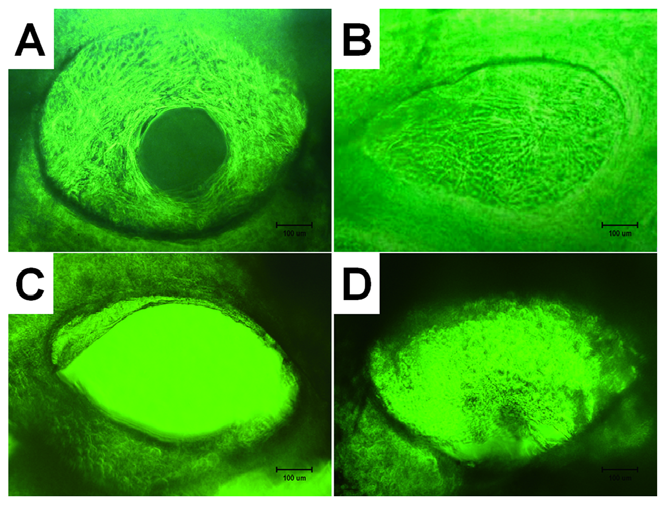
Phase-contrast photomicrographs (×100) of cell-scaffold constructs after in vitro culture for 12 d; (A) group A (dynamic seeding and dynamic culture), (B) group B (hydrogel-assisted seeding and static flask culture, (C) group C (static seeding and static flask culture, control group), and (D) group D (hydrogel-assisted seeding and dynamic culture). Bar lengths are 100 um.

In group A (dynamic seeding followed by dynamic culture), after culture for 8 h, a small number of cells were observed on the surface of the pores in the scaffolds. Then, the cells gradually increased in number and became uniformly distributed on the surface of the pores. After culturing for 5 days, the cells started to produce ECM, the cells and ECM were both uniformly distributed (Fig. 2 A). In group B (hydrogel-assisted seeding followed by static culture), the cell-laden gel filled most pores in scaffold after seeding immediately and the resulting constructs remained almost unchanged during subsequent culture (Fig. 2 B). In group C (static seeding followed by static culture, control group), the seeding suspension rapidly penetrated the DBM scaffolds after seeding and reached the bottom of the wells. Two hours after seeding, a large number of spindle-like cells appeared at the well bottom. After 5 days of subsequent culture, the cells in the scaffolds started to produce extracellular matrix (ECM) resembling spider webs. The cells and ECM were distributed non-uniformly (Fig. 2 C) at this time point. In group D (hydrogel-assisted seeding followed by dynamic culture), the seeded cells were carried by the fibrin gel and filled most pores in the scaffolds after seeding. The cells were identified by their low refractivity (Fig. 2 D). A small amount of fibrin gel was found on the bottom of the wells. The cell number increased with culture time, and was higher than group C at the same time points.

**Figure 3:**
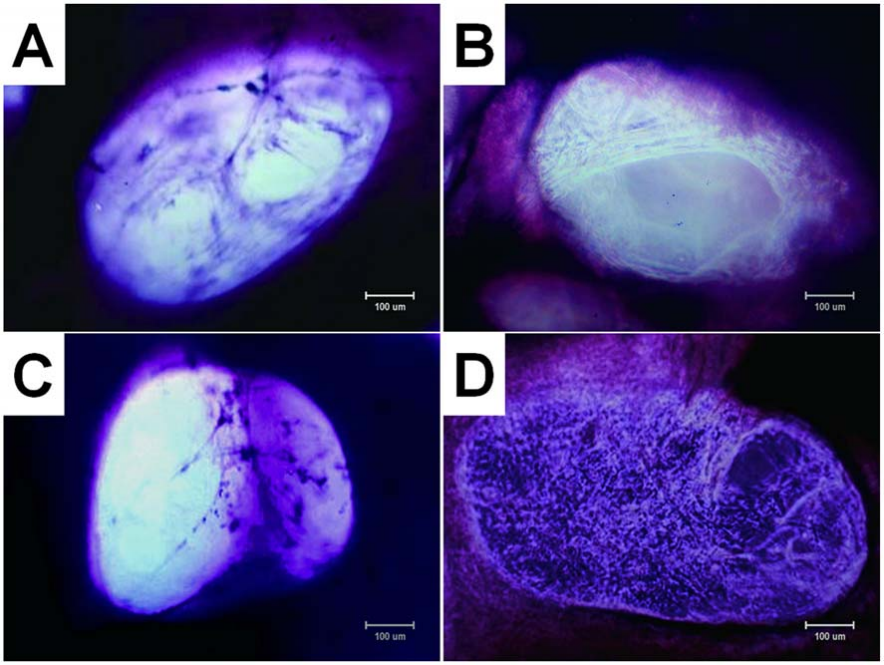
Photomicrographs (×100, methyl violet staining) of cell-scaffold constructs after in vitro culture for 12 d. The number of attached cells and density of extracellular matrix (ECM) fibers in the interior of the scaffold are obvious different among four groups, with group B (B) > group D (D) > group A (A) > group C (C). Bar lengths are 100 um.

The number of attached cells and density of ECM fibers in the interior of the scaffold after 14-day culture are significantly different among four groups. The number of attached cells and density of ECM fibers in group B is most and the cells and ECM were uniformly distributed in the scaffold (Fig. 3 B). The cells in the scaffolds and ECM fibers presented in group C are minimal and non-uniformly scattered in the pores of the scaffolds (Fig. 3 C). Group D (Fig. 3 D) had more cells attached to the pore and ECM than group A (Fig. 3A).

### Seeding efficiency

The seeding efficiency was 32.10±0.72% in group A and 25.24±1.56% in group C, compared with 82.14±1.09% in group B and 81.53±1.37% in group D. Groups B and D were similar (p = 0.396), and the differences between all other group pairs were highly significant (all p<0.01).

### Cell proliferation and osteoblastic differentiation

**Figure 4:**
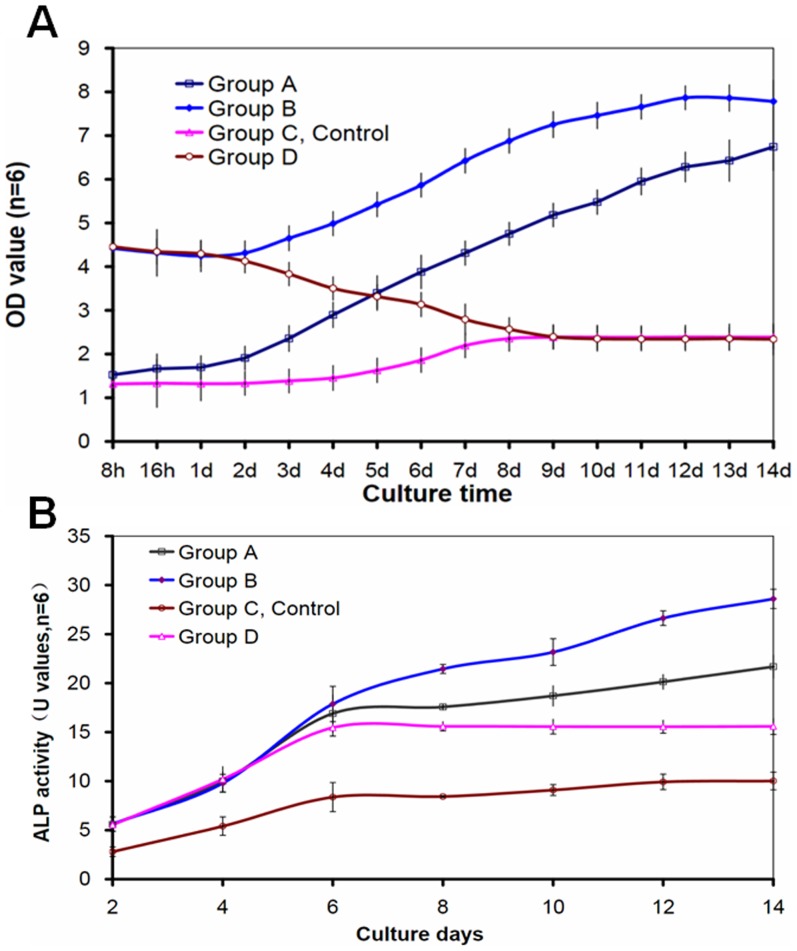
Proliferation of seeded cells in cell-scaffold constructs was detected by cell counting kit-8 (A) and osteoblastic differentiation of seeded cells in cell-scaffold constructs was evaluated by ALP activities (B). The number of cells was increased with culture time except group C. The dynamic culture (groups A and B) showed an obvious ability of promoting proliferation of cells. The ALP activities in all groups increased from day 2 to day 14 (B). The ALP activities in groups A, B, D were statistically higher than that in groups C(p<0.05) from day 4 to day 14. indicates a statistically higher value compared with group C(p<0.05).

In group A, the cell number remained stable during the first 3 days, followed by a continuous increase till day 14. In group B, the cell number was stable during the first 2 days, then started to increase, and attained a plateau on day 12. In group C (control group), the cell number (Fig. 4A) remained stable during the first 4 days in culture, then started to increase, and plateaued on day 8. In comparison, the cell number in group D decreased slightly between 8–24 h in culture, then decreased more rapidly between days 1–8, and remained stable thereafter.

The ALP activities in all groups increased from day 2 to day 6 (Fig. 4B). The activities in groups A and B remained stable thereafter. In comparison, the activities in groups C and D continued to increase, although at lower levels and slopes.

### SEM of cell-scaffold constructs

**Figure 5:**
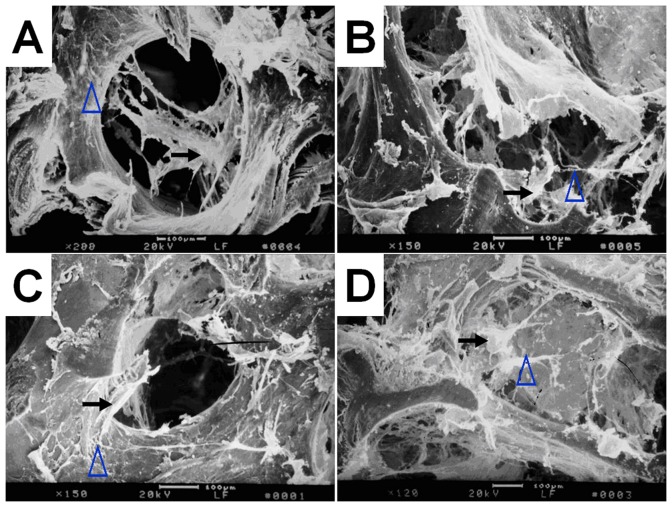
Scanning electron micrographs of cell-scaffold constructs after in vitro culture for 12 days. The attached cells and extracellular matrix (ECM) fibers presented on the scaffolds in group B (B) and group D (D) are significantly outnumber those in group A (A) as well as group C (C).Bar lengths are 100 um. The black arrows indicate cells and the blue arrows indicate ECM fibers.

SEM revealed similar results to optical microscopy. The number of cells and density of ECM fibers in group B (Fig. 5B) is most, then group D (Fig. 5 D), followed by group A (Fig. 5A) and group C (Fig. 5 C).

### X-ray radiography

**Figure 6:**
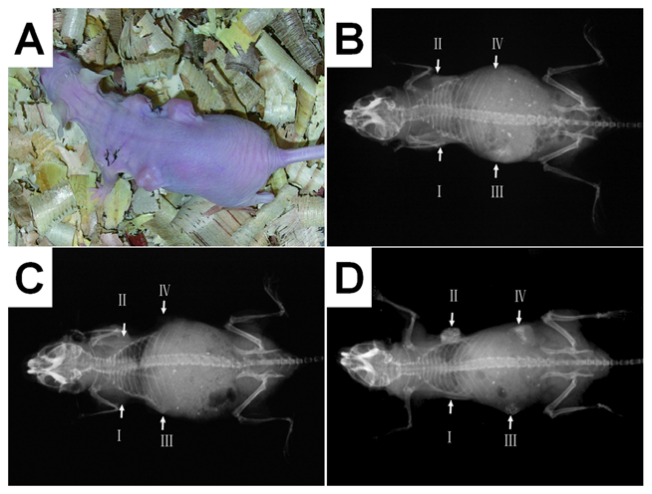
Nude mice subcutaneous implantation model for the evaluation of osteogenic activity; (A) a photograph showing a nude mouse with four implants; (B) a radiograph 4 weeks after implantation; (C) a radiograph 8 weeks after implantation; (D) a radiograph 12 weeks after implantation. The radiographic densities of the implants increased from week 4 to week 12. The osteogenesis of implants was not clear at weeks 4 and 8 postoperative. It was not until 12 weeks postoperative that the imagings of implants in the radiographs were clearly observed. At week 12, implant II clearly showed increased density indicating calcification.

The radiographic densities of all implants increased from week 4 to week 12 (Fig. 6). Implant II(hydrogel-assisted seeding of 2×10^7^/ml hMSCs, followed by dynamic culture for 12 d) showed substantially higher density than the other implants, and implant I (cell-free DBM scaffold) had the lowest densities at both time points. At week 12, implant I showed a slightly higher density compared with the host soft tissue, while implant II clearly showed increased density indicating calcification. The implants III (hydrogel-assisted seeding of 1×10^8^/ml hMSCs without further *in vitro* culture) and IV (hydrogel-assisted seeding 2×10^7^/ml MSCs followed by static culture for 12 d) also showed signs of calcification, but substantially weaker than that in implant II.

### Wet weight and bone mineral density

**Figure 7:**
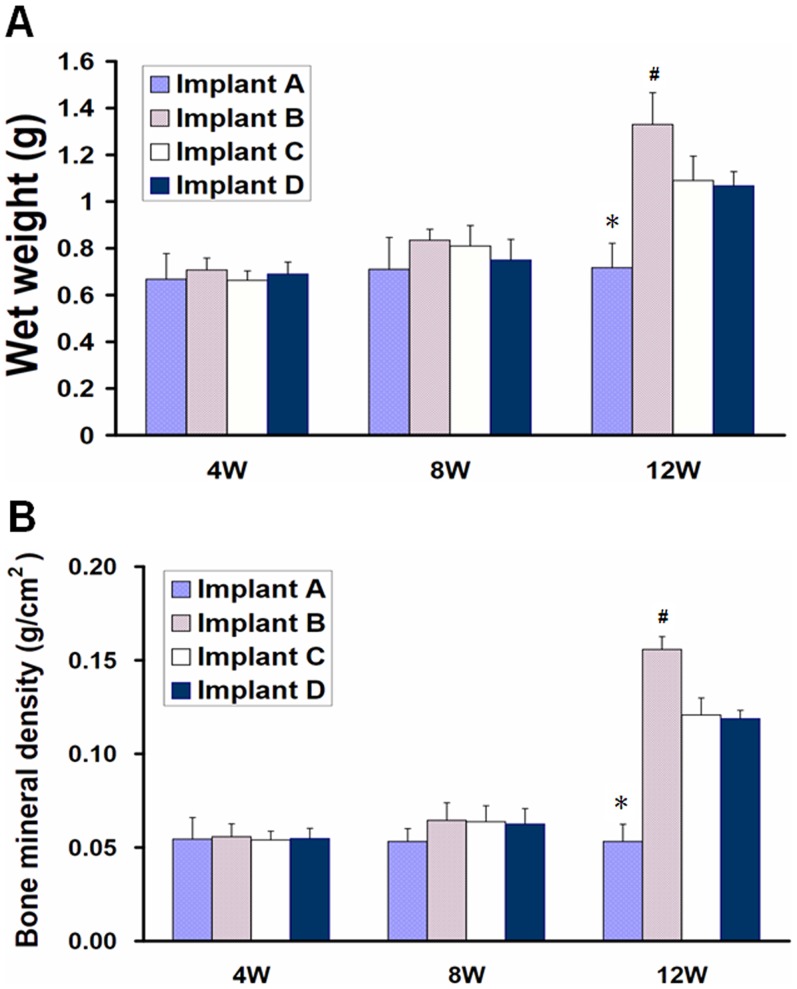
Wet weight and bone mineral density of implants after subcutaneous implantation in nude mice. At 12 weeks postoperative, implant in group II showed higher wet weight (A) and bone mineral density (B) than that in other groups(p<0.05). *indicates a statistically significantly lower value compared with other implants; # indicates a statistically higher value compared with other implants.

Twelve weeks after implantation, implant I showed a significantly lower wet weight compared with other implants (all p<0.01). Moreover, the wet weight of implant II was statistically higher than the implants III (p = 0.008) and D (p = 0.004). Implants III and IV were similar (p = 0.770) (Fig. 7A).

Twelve weeks after implantation, implant II showed a significantly higher bone mineral density than all other implants (all p<0.01). The bone mineral density of implant I was significantly lower than the other implants (all p<0.01). Implants III and IV were similar (p = 0.741) (Fig. 7B).

### Histology of retrieved implants

**Figure 8:**
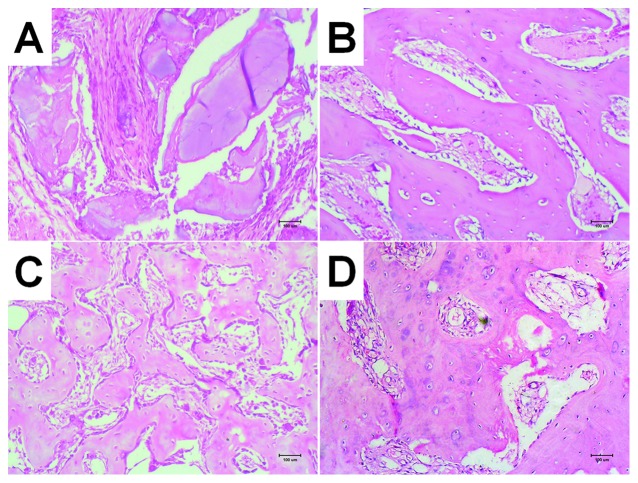
HE staining of ectopic bone formation in nude mice at 12 weeks (×100), Implant I can be seen partially degraded DBM stand, surrounded by fibrous connective tissue replaced; Implant II showed more mature bone structure of a small beam than other groups; both Implant III and IV showed small beam structure of bone with some cartilage-like structure partially visible, bone formation maturity lower than Implant II.

Twelve weeks after implantation, implant I (Fig. 8A) showed partial degradation of DBM scaffold and replacement by fibrous connective tissues around the periphery. Implant II (Fig. 8B) showed relatively mature bone trabeculae but no chondroid tissues. Implant III (Fig. 8C) showed less mature bone trabeculae than implant II, in addition to chondroid structures in a few locations. Implant IV (Fig. 8D) showed new bone trabeculae that were less mature than those formed in implants II and III; transformation of chondroid tissue to immature bony tissue was also locally observed.

## Discussion

In the present study, we evaluated the effects of seeding methods on seeding efficiency and initial cell density for constructing tissue-engineered bone. Compared with other synthetic bone substitutes, tissue-engineered grafts generally have superior osteogenic activities because of the incorporation of seeded cells. Various factors can influence the osteoblastic differentiation of marrow stromal cells in tissue engineering scaffolds during cultivation, including the density and spatial distribution of the seeded cells in the scaffolds [1], [2], [4].

Seeded cells are commonly seeded in scaffolds by static infiltration. Although convenient, this method can attain only limited cell density. Under the action of gravity force, the seeded cells are easily detached from the scaffold and became concentrated at its bottom side, thus resulting in loss of cells. Various methods have been used to promote cell penetration and minimize cell detachment [20], [21], such as the use of negative pressure and magnetic field. Although effective to varying degrees, these methods cannot substantially increase the initial cell density in the scaffold. Recent studies found that RWVBs can produce a simulated microgravity environment to allow cells to diffuse and become uniformly distributed in the interior of scaffolds [9], [22].

Hydrogels have been combined with seeded cells to construct grafts for the repair of cartilage as well as bone [13]. Hydorgels alone, however, are not satisfactory for constructing bone grafts because of their poor strength and limited bone conductivity. Despite this major disadvantage, hydrogels may improve the adhesion between seeded cells and the scaffold [13].

In this study, we compared the seeding efficiency and initial cell density resulting from three seeding methods: fibrin hydrogel-assisted seeding, hydrodynamic seeding (simulated microgravity in RWVB), and the simple static infiltration. Microscopy, cell counting, and viability assays showed that fibrin hydrogel-assisted seeding generated a significantly higher seeding efficiency and initial cell density than the other two methods. The improvement can increase the utilization of seeded cells and is expected to increase the osteogenic activity of the resulting grafts.

Fibrin glue has been clinically confirmed to be safe, biocompatible, and fully absorbable within two weeks [23]. A recent clinical study used fibrin as a carrier for chondrocytes to treat cartilage defects and obtained positive results [24]. The fibrin glue used in this study was a mixture of fibrinogen, thrombin, factor XIII, and calcium salt. Fibrinogen is a major plasma protein (350 kDa) that stimulates proliferative signals by serving as a scaffold to support the binding of growth factors and to promote the cellular responses of adhesion, proliferation, and migration during wound healing [25]. Thrombin is an enzyme that converts soluble fibrinogen into insoluble fibrin between 10 and 60 seconds and acts as a tissue adhesive [26]. Factor XIII, which exists in the fibrinogen component of the glue, cross links and stabilises the clot's fibrin monomers [27]. These glue contents in mixture formed an efficient cross-linking network that could capture MSCs rapidly and promote the cell attachment and proliferation. Therefore, higher seeding efficiency was obtained in fibrin hydrogel-assisted seeding groups.

We further identified the effect of hydrodynamic culture on cell proliferation and differentiation *in vitro*. There is still no consensus on whether tissue-engineered bone grafts need to be cultured *in vitro* before implantation. Many studies have suggested that *in vitro* culture can allow the seeded cells to stably adhere on the scaffold and, thereby, prevent their detachment, migration, or death resulting from changes of microenvironment [3], [4], [28]. Wang et al, however, suggested that the *in vivo* condition should be optimal for the growth, differentiation, and function of cells. In contrast, *in vitro* cultured constructs may be structurally unstable, mechanically weak, and subject to changes in tissue structure and type [29]. In an attempt to combine the advantages of pre-implantation culture and *in vivo* microenvironment, some studies also explored ectopic implantation to engineer mature, vascularized bone grafts [30]. These “*in vivo* engineered” grafts were found to have superior osteogenic activities, but the technique involves a long *in vivo* culture and additional damage to the patient.

Recent development of bioreactor techniques has made it possible to better simulate the *in vivo* microenvironment, promote mass exchange, and create appropriate mechanical stimuli. These improvements may be used to produce more mature and bioactive tissue-engineered grafts [31]. In tissue engineering of grafts, the supply of nutrients and removal of metabolic wastes is more difficult than in conventional cell culture. The mass transport in the common static culture method relies on the concentration gradient and is thus inefficient [32]. As a result, cells typically do not survive well in the center of the graft and in some cases even undergo necrosis to form voids [33]. This has severely limited the size of grafts that can be obtained by tissue engineering [34]. An appropriately designed bioreactor may provide hydrodynamic conditions to promote mass transfer, stimulate stem cells to differentiate into osteoblasts, and thus overcome this disadvantage.

In this study, we found that when comparing static and hydrogel-assisted seeding, the statically cultured cell-scaffold constructs achieved lower plateau values. In comparison, regardless of the initial cell densities, the dynamically cultured constructs showed continued increase in cell density and became approximately two times higher than the statically cultured grafts. Furthermore, with a higher seeding efficiency and cell density by the hydrogel-assisted seeding, group B achieved plateau earlier than the group A. The ALP activities of the constructs (Fig. 3A) followed the order of: group B>group A>group D>group C, consistent with the trend of cell number between days 6–14 (Fig. 3B). These findings suggest that hydrogel-assisted seeding followed by hydrodynamic culture can substantially increase the initial seed cell density in constructs, achieve a higher cell density earlier than static culture, and is the optimal one among the four methods studied here.

The favourable effect of hydrodynamic culture may be attributed to three factors. First, the vortex in the bioreactor generated fluid flow in the construct, which enhanced mass transfer and improved the cell distribution [4], [7]. A computational analysis suggested that sufficient flow fluid can be generated in porous scaffolds despite being partially sealed with a material similar to fibrin. Second, the shear stress resulting from the fluid flow may have simulated the seeded cells to differentiate, mature, produce extracellular matrix, and calcify [7]. Third, the hydrodynamic condition might promote cell-cell, and cell-matrix interaction and signal communication, which enhanced their autocrine/paracrine activities and maintained their differentiation [4], [22].

In this study, we also observed that osteogenic activity could be influenced by the initial cell number and in vitro culture methods. Ectopic osteogenesis in nude mice is a widely used method for evaluating the performance of bone substitutes. Moreover, subcutaneous implantation is a challenging model for the implants because of the lack of osteoblast progenitors in the implantation area. Twelve weeks after implantation into the subcutaneous pocket, implant I (cell-free DBM) was filled mainly by soft tissues and showed only slight increase in radiographic density, indicating its lack of osteogenic activity in this site. Implant II showed the highest osteogenic activity according to radiography, histology, wet weight, and bone mineral density. This implant was seeded by the hydrogel-assisted method (2×10^7^ cells/ml, 0.05 ml), followed by hydrodynamic culture for 12 days to achieve the plateau cell number and, hypothetically, the best osteogenic activity. Its superior performance confirmed that the combination of hydrogel-assisted seeding and hydrodynamic culture is a promising protocol for tissue-engineering bone grafts.

Implant III showed an intermediate osteogenic activity between the implants I and II. This implant was seeded with the same number of hMSCs as implant II by the hydrogel-assisted method, and was immediately implanted without *in vitro* culture. Therefore, a comparison between implants III and II demonstrated that the *in vitro* culture increased the osteogenic activity of implants. The increase may be attributed to several aspects. The *in vitro* culture increased the number of seeded cells, and allowed the cells to adhere more stably to the scaffold and thus prevented their detachment after implantation. The cells might also rearrange in order to more effectively interact and communicate with each other [4], [22]. Additionally, the cells might produce extracellular matrix and osteogenic factors during the *in vitro* culture, which accelerated the subsequent osteogenesis in the subcutaneous pocket.

Similarly, implant IV also showed lower osteogenic activity than implant II. Compared with implant II, implant IV was seeded with the same number of cells but statically cultured *in vitro* before implantation. Its inferior performance may be primarily attributed to its lower cell number as a result of the static culture, which lacked mechanical stimulation for the cells to proliferate and differentiate [11].

In summary, both *in vitro* and *in vivo* results suggest that hydrogel-assisted seeding can significantly increase the seeding efficiency and the initial cell density in the cell-scaffold construct. A subsequent hydrodynamic *in vitro* culture can significantly increase the plateau cell density. Correspondingly, bone grafts produced by the combination of these two methods can achieve the highest osteogenic activity. These findings can have a significant bearing in clinical applications and in optimizing tissue engineering strategy.
